# Elimination within reach: A cross-sectional study highlighting the factors that contribute to persistent lymphatic filariasis in eight communities in rural Ghana

**DOI:** 10.1371/journal.pntd.0006994

**Published:** 2019-01-04

**Authors:** Corrado Minetti, Edward J. Tettevi, Frank Mechan, Joaquín M. Prada, Bright Idun, Nana-Kwadwo Biritwum, Mike Yaw Osei-Atweneboana, Lisa J. Reimer

**Affiliations:** 1 Department of Vector Biology, Liverpool School of Tropical Medicine, Liverpool, United Kingdom; 2 Department of Environmental Biology and Health, Council for Scientific and Industrial Research Water Research Institute, Accra, Ghana; 3 Mathematics Institute, University of Warwick, Coventry, United Kingdom; 4 School of Veterinary Medicine, Faculty of Health and Medical Sciences, University of Surrey, Guildford, United Kingdom; 5 Neglected Tropical Diseases Programme, Ghana Health Service, Accra, Ghana; RTI International, UNITED STATES

## Abstract

**Background:**

Despite the progress achieved in scaling-up mass drug administration (MDA) for lymphatic filariasis (LF) in Ghana, communities with persistent LF still exist even after 10 years of community treatment. To understand the reasons for persistence, we conducted a study to assess the status of disease elimination and understand the adherence to interventions including MDA and insecticide treated nets.

**Methodology and principal findings:**

We conducted a parasitological and epidemiological cross-sectional study in adults from eight villages still under MDA in the Northern Region savannah and the coastal Western Region of the country. Prevalence of filarial antigen ranged 0 to 32.4% and in five villages the prevalence of night blood microfilaria (mf) was above 1%, ranging from 0 to 5.7%. Median mf density was 67 mf/ml (range: 10–3,560). LF antigen positivity was positively associated with male sex but negatively associated with participating in MDA the previous year. Male sex was also associated with a decreased probability of participating in MDA. A stochastic model (TRANSFIL) was used to assess the expected microfilaria prevalence under different MDA coverage scenarios using historical data on one community in the Western Region. In this example, the model simulations suggested that the slow decline in mf prevalence is what we would expect given high baseline prevalence and a high correlation between MDA adherence from year to year, despite high MDA coverage.

**Conclusions:**

There is a need for an integrated quantitative and qualitative research approach to identify the variations in prevalence, associated risk factors and intervention coverage and use levels between and within regions and districts. Such knowledge will help target resources and enhance surveillance to the communities most at risk and to reach the 2020 LF elimination goals in Ghana.

## Author summary

Lymphatic filariasis (LF) is a mosquito-borne disease and a leading cause of disability and chronic morbidity worldwide. Despite the progress achieved so far in stopping LF transmission by treating the affected communities with specific drugs over several years, areas where lymphatic filariasis persists still exist. Understanding the reasons behind this is pivotal to both reach and sustain elimination. We investigated the factors associated with filariasis persistence in various communities still under drug treatment from two regions of Ghana. We reported high variability in disease burden, adherence to drug treatment and mosquito net use between regions and communities. LF infection was associated with men and not taking the drugs, and men were also less likely to take treatment. Using mathematical modelling, we showed that slight increases in treatment coverage will accelerate elimination. Our findings highlight the reasons for LF persistence and provide guidance on how to successfully achieve elimination by refining drug treatment distribution and mosquito control interventions more tailored to individuals and communities. We also demonstrated the value of using field-collected data in mathematical models to assess the current status of disease elimination and to identify the gaps in control interventions.

## Introduction

Lymphatic filariasis (LF) is a mosquito-borne disease caused by the filarial nematodes *Wuchereria bancrofti*, *Brugia malayi* and *Brugia timori*. LF is a leading cause of disability and chronic morbidity worldwide with 1.38 billion people living in endemic areas globally, and it usually manifests in adulthood with lymphedema, elephantiasis and hydrocele [1]. LF creates a considerable physical, socio-psychological and economic burden to the affected individuals and communities. In the African region, where up to 464 million people in 33 countries live in endemic areas [1], LF is caused by *W*. *bancrofti* and is most commonly transmitted by mosquitoes of the genus *Anopheles* (in rural areas across the whole continent) and *Culex* (in urban areas of Eastern Africa) [2]. The Global Programme to Eliminate Lymphatic Filariasis (GPELF) was established in 2000 by the World Health Organization (WHO) with the goal to eliminate LF as a public health problem by the year 2020 [3]. The mainstay of the elimination program is mass drug administration (MDA) of the anthelminthic drugs albendazole and either ivermectin in areas co-endemic for onchocerciasis or diethylcarbamazine. These drugs kill the microfilaria (mf), the juvenile stage which is responsible for the transmission to the mosquito, and repeated annual drug treatment of the at-risk population is expected to reduce the prevalence of infection below a threshold (1% mf prevalence) under which transmission cannot be sustained [4]. In areas classified as endemic for LF after mapping, WHO recommends at least 5 years of MDA with 65% coverage of the total population [5]. Once mf prevalence falls below 1%, a transmission assessment survey (TAS) is implemented to determine the parasite antigen prevalence in 6–7 year old schoolchildren. In areas where *Anopheles* spp and *Culex* spp are the primary vectors, if the detected antigenemia in school children is <2% then MDA can be stopped. To avoid recrudescence of LF, the TAS should be repeated at years 2–3 and 4–6 after stopping MDA [5].

Since the year 2000, Ghana has been one of the first countries implementing MDA for LF elimination and it has made tremendous progress in the program scaling up in the past 15 years [6]. Before the inception of MDA, the prevalence, distribution and clinical characteristics of LF in the country were characterized extensively for the first time in 1994 with a national survey [7]. Variation in the prevalence of microfilaria ranged from 0 to 20%, with adults 40 years of age and older being more affected, and the disease was found to be more concentrated in the northern and southern savannah areas. A successive study using antigen prevalence data revealed that LF was endemic in 49 of the 110 country districts which later increased to 98 of a total of 216 following re-demarcation of the districts, and confirmed the presence of the two major transmission zones in the north and along the southwest coast [8]. In Ghana, as in most of West Africa, LF is a rural disease transmitted by members of the *Anopheles gambiae* and *A*. *funestus* complexes [9], although recently also *Mansonia* spp mosquitoes have been incriminated as potentially effective vectors [10].

After numerous rounds of MDA and by 2015, 83 districts in the country had passed the TAS, treatment had stopped and the districts are now under post-MDA surveillance [6]. However, despite over 10 years of treatment with above 65% reported coverage, 15 districts are still undergoing annual MDA due to an observed mf prevalence of >1% [6]. Such districts are located mostly on the coast of the Western Region and in the Northern Region savannah, within the historically high prevalence zones [8]. A high baseline microfilaria prevalence is an important factor in explaining the persistence of infection after several years of MDA in such “hotspot” areas [11]. It is unclear whether other unique transmission dynamics or socioeconomic factors in these areas have compromised the efficacy of MDA and are contributing to the slower than expected decline in mf prevalence.

Mathematical models have played a major role in understanding the dynamics of LF transmission and the prospects for its elimination [12,13]. Currently, three major models have been developed (EPIFIL, LYMPHASIM and TRANSFIL) which differ in being either individual or population-based, the number of parameters and how infection aggregation or drug and vector control are incorporated [14]. Although models showed that elimination can be achieved using annual MDA alone [12], it is also clear that a sustained reduction in the mosquito biting rates using vector control can accelerate elimination, resulting in an important reduction of annual MDA rounds needed to reach the elimination threshold [15]. More importantly, it is clear that factors such as baseline prevalence, treatment coverage and systematic adherence, coupled with spatial heterogeneities in LF transmission can greatly affect the duration of MDA and ultimately the prospects of elimination [12].

In order to achieve and sustain the 2020 LF elimination goals [3], it is important to better understand the potential causes of the observed heterogeneity in infection prevalence after several years of MDA. The efficiency of the MDA program in decreasing parasite prevalence and stopping transmission, as well as the capacity of assessing whether the program works as expected, can be greatly influenced by a variety of factors related to MDA coverage and individual adherence [16], the role of community drug distributors and health workers and data reporting and quality [17,18].

Furthermore, the use of long-lasting insecticidal nets (LLINs) and other vector control interventions have been shown to play an important role in reducing transmission and accelerating elimination of LF [15,19]. Since LF and malaria in Africa are transmitted by the same anopheline vectors, interventions aimed at controlling and eliminating malaria can have a significant impact on LF [20]. Gender differences in the perception and use of LLINs must also be considered [21], and entomological factors, such as local vector diversity, abundance, biting rates and vector competence play an important role in the prospects of LF elimination [8].

The aim of our study was to identify factors that have contributed to persistent LF infection in two of the ‘hotspot’ regions of Ghana still undergoing MDA and to provide guidance to the national LF programme. We examined village level LF prevalence, sociodemographics and MDA and LLIN coverage and use in adults in eight communities. In addition, we used the stochastic individual-based TRANSFIL model to determine whether the currently observed LF prevalence is higher than would be predicted given the available coverage data and baseline prevalence. The implications of our findings on the continuing transmission of LF in these communities and the proposed refinements needed for the elimination program are discussed.

## Methods

### Study communities

**Fig 1:**
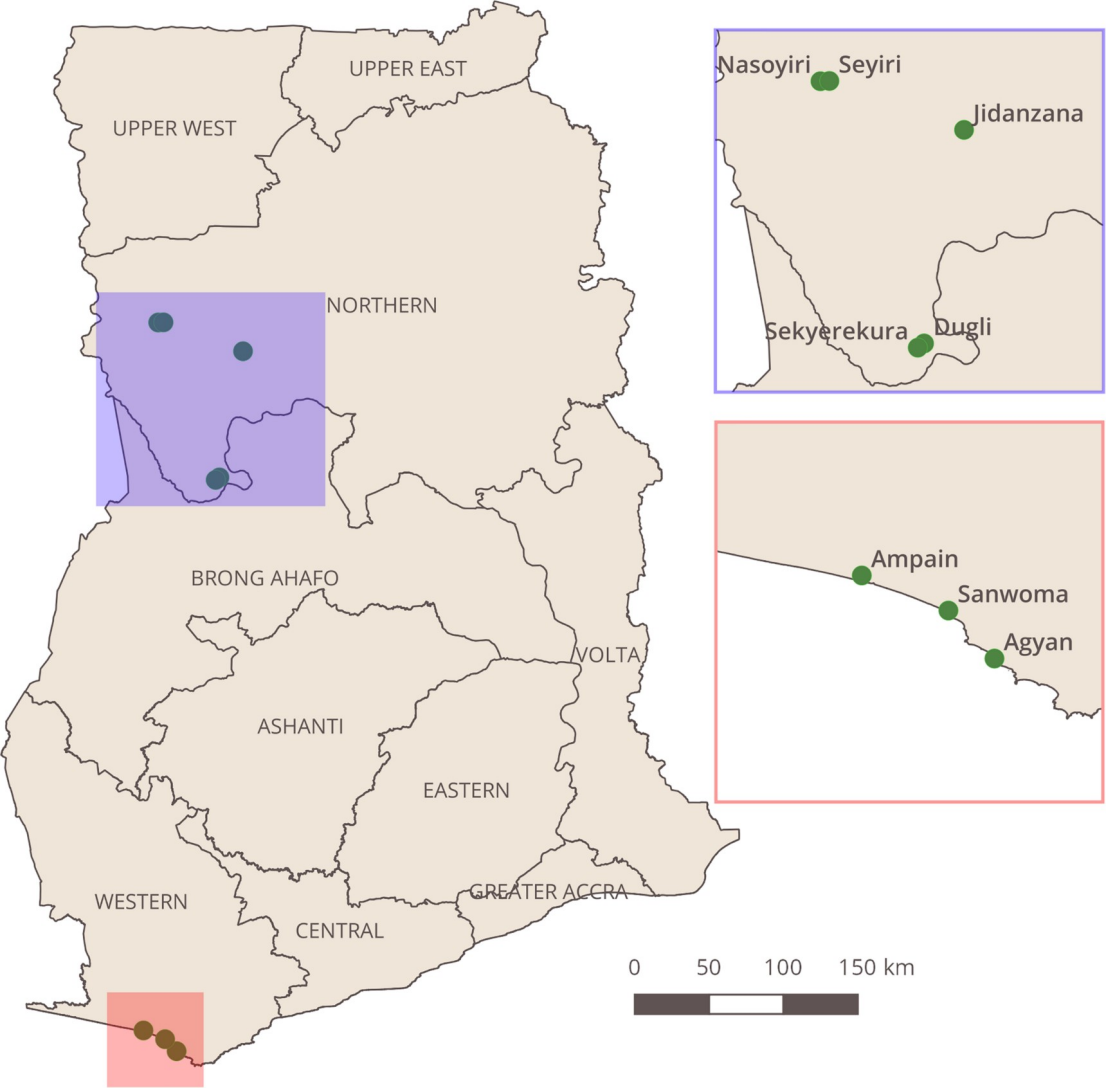
Map of Ghana showing the eight study villages. This map was created using QGIS version 2.18.

The study was conducted in eight rural communities from two regions of Ghana where MDA is still ongoing (Fig 1). The villages of Agyan (Nzema East district), Sanwoma and Ampain (Ellembelle district) were selected from the Western Region. The villages of Dugli and Sekyerekura (Bole district) and Jidanzana, Nasoyiri and Seyiri (Gonja West district) were selected from the Northern Region. Surveys took place in May 2016 in the Western Region and in March 2017 in the Northern Region, prior to annual MDA. The environmental conditions and anopheline vectors species composition greatly differ between the two regions. In the northern drier savanna *A*. *gambiae* s.s, *A*. *arabiensis* and *A*. *funestus* dominate, whereas in the western region coast *A*. *gambiae* s.s. and *A*. *melas* are the major vectors [9,22]. Villages were chosen from districts where MDA was still occurring due to pre-TAS surveys conducted in 2012 and 2014 showing a microfilaria prevalence of >1% (source: National Neglected Tropical Diseases Programme, Ghana Health Service). Following pre-TAS failure, these areas were designated as ‘hotspot districts’ and resources were dedicated to implementation activities to support training, supervision and social mobilisation. In all villages, all adult male and female residents 18 years of age and above were invited to take part in the survey by convenience sampling following a public announcement by the village leader. Participants were asked about LF program knowledge, MDA adherence, frequency of participation, bednet ownership and use.

### Parasitological and epidemiological data collection

The presence of *W*. *bancrofti* adult worm antigen was determined in consenting participants by the Filariasis Test Strip (Alere Inc.,Waltham, USA) immunochromatographic test, using 75 μl of finger-prick blood. A further 2 ml of venous blood were collected from consenting FTS-positive participants between 2200h and 0100h for the detection of microfilariae, using the acetic acid fixation and counting chamber method [23]. Following microscopic analysis, the number of microfilariae per millilitre (ml) of blood was calculated. For all consenting participants, a questionnaire was used to collect age, sex as well as knowledge of the LF elimination program, MDA adherence including the number of previous MDA rounds taken, and LLIN ownership and use. Data were collected on tablets equipped with the Open Data Kit (ODK) application.

### Descriptive statistics and multivariate modelling

Data analysis was performed using the IBM SPSS Statistics (version 22) software and the R programming language (version 3.3.3). Descriptive statistics were calculated for infection prevalence, knowledge of the LF elimination program, MDA participation and LLIN ownership and use overall and by region, communities and sex separately. Generalised Linear Mixed Models (GLMMs) (lme4 version 1.1–13) with stepwise regression were used to identify factors significantly associated with LF antigen positivity and MDA adherence. The model selection process used stepwise regression, the maximally complex model with all fixed variables and all two-way interactions was fit then the significance of each variable was investigated using log-likelihood ratio tests (LRTs). Only variables which contributed a statistically significant (*p*<0.05) increase to the explanatory power of the model were retained. The final model included only the explanatory variables that LRTs indicated were significant.

The region within the country where the participant lived (“Region”) was fit to all models as a fixed categorical effect (“Western” = western region; “Northern” = northern region). The age of participants in years (“Age”) was fit to the models as a fixed continuous effect (18–97). The sex of the participants (“Sex”) was fit to the models as a fixed categorical effect (“Male” = male participant; “Female” = female participant). LLIN use (“LLIN”) was fit as a fixed categorical effect (“Yes” = slept under an LLIN the night before; “No” = did not sleep under an LLIN the night before). To account for variation between individuals and villages, the ID of the participant and the ID of the village in which they lived were each included in the models as categorical random effects.

### Model of LF antigen prevalence

To investigate variations in the presence of filarial antigens, a model of antigen positivity was fit to the data. The response variable (“MF_Pos”) was the presence or absence of the filarial antigen (1 = present; 0 = absent). This model tested how filarial antigen positivity varied by MDA participation, LLIN use, region, age, and sex. Participation in MDA in the previous distribution round (“MDA”) was fit to the model as a fixed categorical effect (“Yes” = taking MDA the year before; “No” = not taking MDA the year before).

### Model of MDA participation the previous year

To investigate variations in participation in MDA, a model of MDA participation was fit to the data. The response variable (“MDA”) was whether or not the respondent reported they had taken MDA the year before (1 = participated in MDA; 0 = did not participate in MDA). This model tested how MDA participation varied by LLIN use, region, age and sex.

### Stochastic individual-based model (TRANSFIL)

Available data on the community of Agyan mf prevalence in the year 2012 (9%) and reported MDA coverage for the year 2016 (80%) (source: National Neglected Tropical Diseases Programme, Ghana Health Service) were used in conjunction with the data collected in this study (mf prevalence, estimated MDA adherence and bednet coverage) to assess the current status of LF elimination in this community using the stochastic individual-based TRANSFIL model [13]. An online version of this model is available [24]. The data from Agyan on adherence to multiple rounds of treatment were fitted to a previously developed model [25]. The observed mf prevalence in 2012 was used to calibrate the model, while the mf prevalence in 2016 was used to assess which MDA coverage better reflects the prevalence reduction observed from 2012 to 2016: 500 simulations were selected at baseline (2012) and run forward in time with *Anopheles* spp. as the dominant vector, with 27% LLIN coverage and annual treatment with ivermectin and albendazole. More details about model parameters and fitting are given in the S1 Supporting Information.

### Ethical clearance

This study was approved by the Ethics Committees of the Liverpool School of Tropical Medicine, United Kingdom (Research Protocol 15.042 COUNTDOWN Integrated Control Strategies to Eliminate Lymphatic Filariasis in Ghana), and the Council for Scientific and Industrial Research, Accra, Ghana. Before enrolment of participants, members of the research team met with the district health officials and community leaders to explain the purpose of the study. Community meetings were held to explain the purpose of the study to all residents, and further details were explained to all participants before they signed an informed consent form. Participants were informed of their test result by a district health official and all community members were encouraged to prevent transmission through bednet usage and MDA participation. Community averaged antigen and microfilaria prevalence was shared with the LF program.

## Results

### LF antigen and microfilaria prevalence

The prevalence of *W*. *bancrofti* antigen determined by FTS and the estimated microfilaria prevalence (based on the proportion of FTS+ individuals that tested positive for mf), alongside available data on the same sub-districts and communities from previous years, are shown in Table 1. Antigen prevalence varied between the two regions and communities (from 0 to 32%), and the estimated prevalence of microfilaria was above 1% in five villages. Overall, both the antigen and microfilaria prevalence were higher in the Northern Region communities. The antigen prevalence by gender and age group in the sampled communities is reported in the S1 Table.

**Table 1:** *W*. *bancrofti* antigen and microfilaria prevalence in the surveyed communities.

				Current study			
Region	District	Community	Previous mf prevalence (%) (year)	No. tested for antigen*	% FTS+ (no.)	Mf+/ tested for mf	Mf prevalence estimate^a^
Western	Nzema East	Agyan	25.4 (1995), 9 (2012), 3.7 (2014)	171	24.6 (42)	2/24	2.0
	Ellembelle	Ampain	n/a	160	8.8 (14)	1/11	0.8
		Sanwoma	4.5 (2014)	176	10.2 (18)	0/15	0
Northern	Bole	Dugli	5.7 (2014)	105	21.0 (22)	5/19	5.5
		Sekyerekura	n/a	58	22.8 (14)	4/13	7.4
	West Gonja	Nasoyiri	n/a	75	20.0 (15)	2/15	2.7
		Seyiri	4.3 (2014)	68	32.4 (22)	1/21	1.5
		Jidanzana	n/a	99	0 (0)	nd	nd

mf = microfilaria; n/a = not available; nd = not done

*12 participants in total had an invalid antigen test after two replicates

^a^population estimate based on mf prevalence in the antigen positive participants multiplied by the proportion antigen positive participants in the community.

In the 15 individuals that tested positive for microfilaria, the median mf density was 67 mf/ml (range 30 to 3,560). The median number of mf/ml was 217 (range 67 to 551) in the Western and 30 (range 10 to 3,560) in the Northern region. The two individuals with the highest mf density were observed in Sekyerekura (1,140 mf/ml) and Dugli (3,560 mf/ml). The median age of microfilaria positive participants was 37 years (range 19 to 80) and 12 (80%) were males. The mf prevalence we observed in the study communities was lower than the prevalence observed in previous years, with available data showing a decrease within two years of continuing MDA (Table 1).

### LF elimination program knowledge and MDA participation

**Fig 2:**
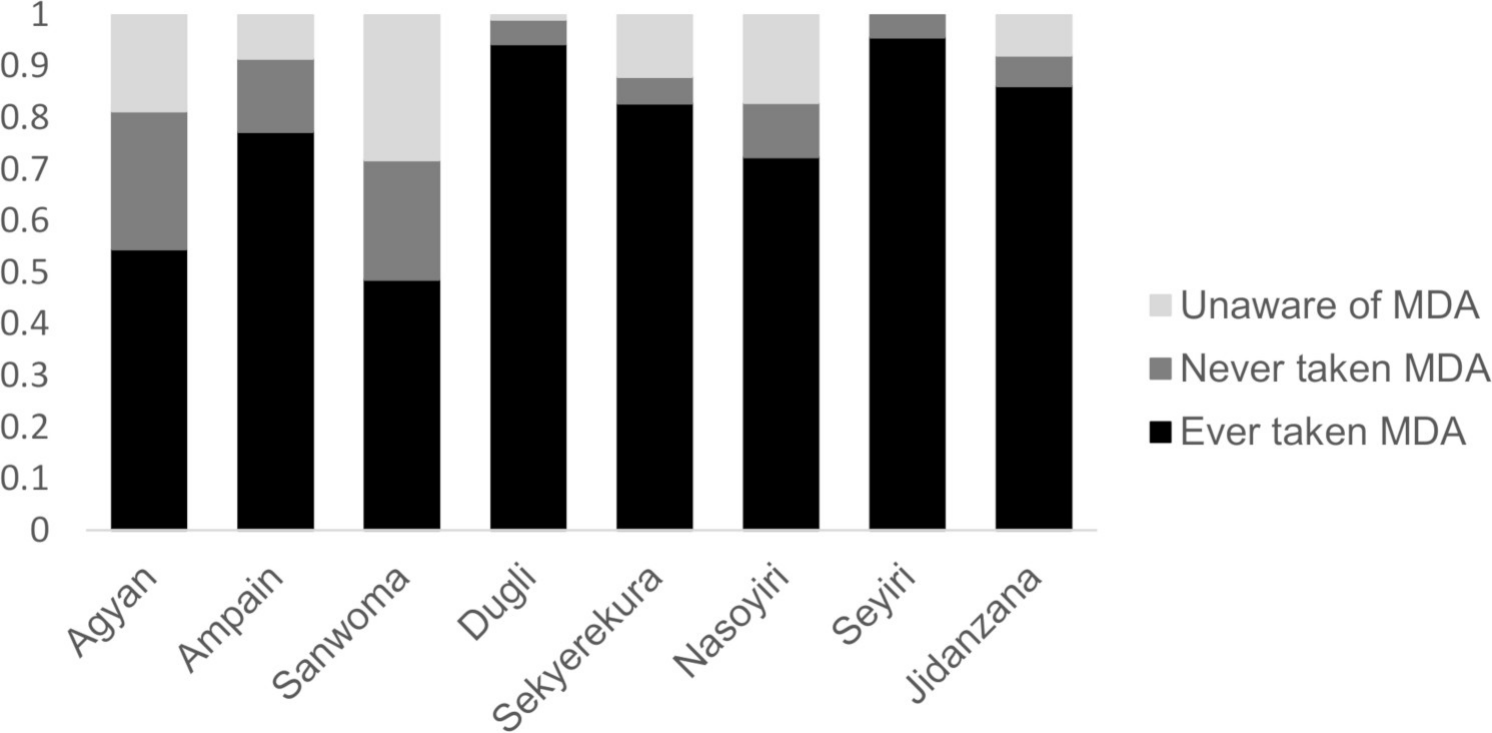
MDA coverage by village. Study participants were first asked whether they were aware of the filariasis drug distribution. If they answered yes they were asked whether they had ever taken part in the MDA and the number of rounds they recalled participating in. The median number of rounds reported was as follows: Agyan (8), Ampain (4), Dugli (3), Jidanzana (4), Nasoyiri (2), Sanwoma (3), Sekyerekura (3), Seyiri (8).

Of the 924 participants that were administered the questionnaire, 797 (86%) knew about the LF elimination program. The level of knowledge varied between communities, but overall it appeared to be higher in the Northern Region communities (93%) than in the Western Region (81%) (Fig 2). No differences in the program knowledge were observed by sex. Of the 797 participants that knew of the LF program and were asked further questions on MDA adherence, 661 (83%) reported taking the drugs for LF MDA at least once. The number of people reporting ever taking the drug was higher in the Northern (93.4%) than in the Western (74%) regions, and similar in females and males (84% versus 81%). Of the 632 people that also reported the year they last took the drugs, 513 (81%) reported taking the drugs during the previous distribution round. The proportion was higher in the Northern (89%) than in the Western region (72%). 32% of total participants were unable to answer the question because they could not recall if or when they had taken the drugs. The LF program reported coverage ranging from 87% to 94% in seven of the study villages in 2016 (data were not available from Sanwoma). Statistics on MDA participation, including the median number of times MDA was taken in the previous distribution rounds, for each surveyed community are reported in Fig 2.

Overall and consistently across communities, the most frequently reported reason for not taking the drugs by the 136 (15% of the total) non-adherent participants that knew about the program was not being present while drugs were distributed (63%) followed by not liking taking pills or tablets or fear of side effects (14%) or being pregnant during distribution (11%). It is possible the further 14% of individuals (n = 126) that did not know about the distribution were also absent during the distribution, although it is possible they participated without knowing the purpose of the MDA. Two out of these 126 participants had moved to the village within the last year, however 88% have resided there for over five years.

### LLIN ownership and use

**Fig 3:**
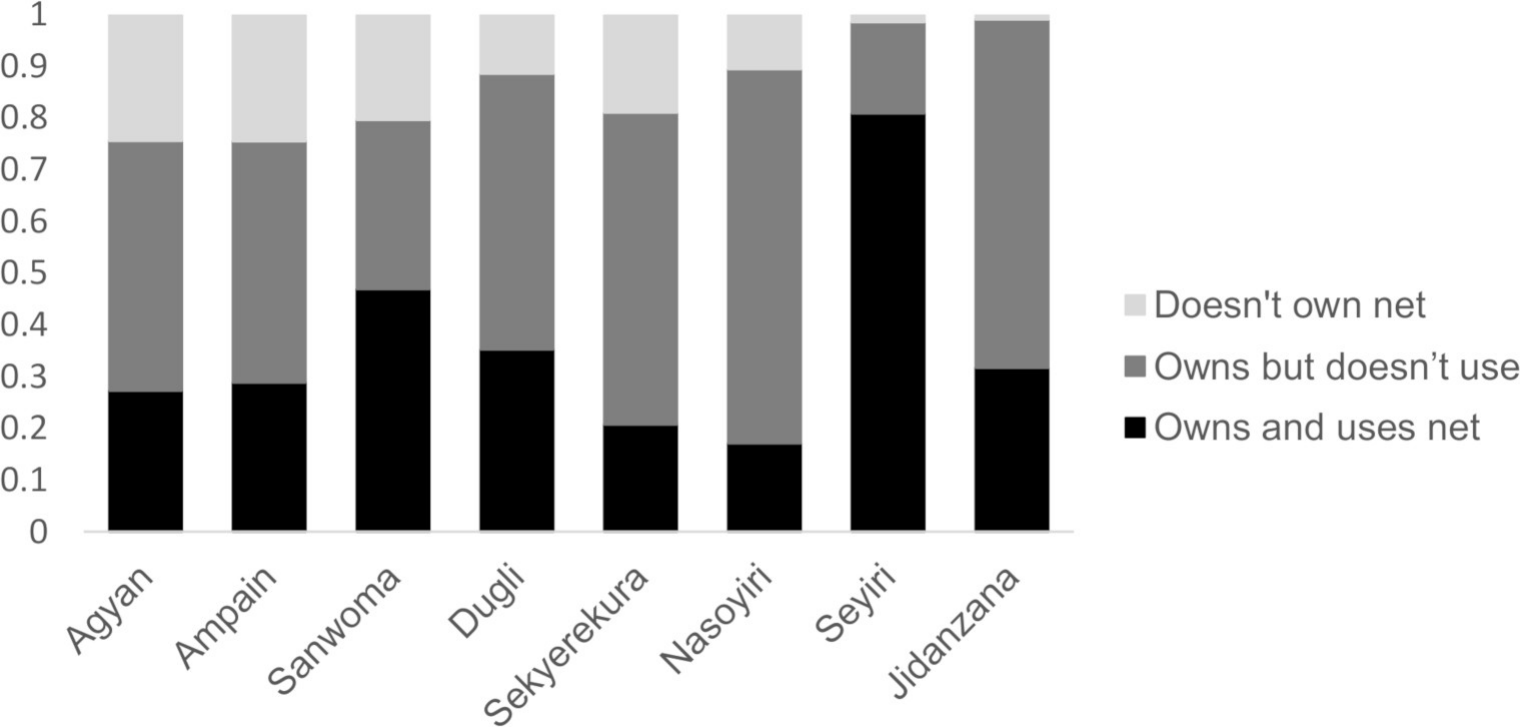
Bednet ownership and use by village. Participants were asked whether they owned a bednet and whether they slept under their net the previous night.

Of the 924 participants that were administered the questionnaire, 772 (84%) reported owning an LLIN. Ownership was higher in the Northern Region (92% versus 73% in the Western Region) and lower in males (78% versus 86% in females). Net ownership was positively correlated with MDA participation ((*χ*2 = 16.9, p<0.001). Overall and consistently across communities, the most frequently reported reasons for not owning a net (152 participants) were related to the mass distribution: people reporting not having been given a net, not being present during distribution or either not being able to find one or afford it, cumulatively accounted for most participants (66%). Other reasons included people which didn’t feel they needed a net or didn’t liking sleeping under it (24%) or because the net was damaged (6.6%). Of the people owning a net, only 337 (42.4%) reported sleeping under it the night before the interview. The use of bednets was low in both regions (40% in the Northern and 45% in the Western region), but was also lower in males (35.5%) than in females (47%). The differences in bednet ownership and use for each surveyed community are shown in Fig 3.

### Predictors of LF antigen positivity

The results from the GLMM on the predictors of LF antigen positivity are shown in Table 2.

**Table 2:** Summary of modelled coefficients for LF antigen positivity.

Response	Parameter	Odds Ratio	Lower 95% CI	Upper 95% CI	*p*-value^a^
Positive for LF antigen	(Intercept)	0.079	0.033	0.187	<0.001
	Sex(Male)	1.978	1.346	2.907	<0.001
	MDA in the previous year (No)	1.620	1.051	2.497	0.029

^**a**^ estimated using Likelihood Ratio Tests

Males were significantly more likely to be positive for the FTS antigen than females (*χ*2 = 13.642, df = 1, p<0.001), with sex a stronger predictor of FTS positivity than any other variable included in the final model (OR = 1.970, Table 2). The prevalence of LF antigen was significantly higher amongst those that did not take MDA in the previous year (*χ*2 = 6.995, df = 1, p = <0.001). The interaction between MDA participation in the previous year and sex was not significant (*χ*2 = 0.669, df = 1, p = 0.413) indicating that there was no evidence of variation in MDA participation between males and females. There was no significant difference in LF antigen positivity between those that slept under an LLIN the previous night and those that did not (*χ*2 = 3.646, df = 1, p = 0.056).

### Predictors of MDA participation in the previous year

The results from the GLMM on the predictors of MDA participation are shown in Table 3.

**Table 3:** Summary of modelled coefficients for MDA participation.

Response	Parameter	Odds Ratio	Lower 95% CI	Upper 95% CI	*p*-value^a^
Participated in MDA the previous year	(Intercept)	0.414	0.151	1.130	0.085
	Age	1.015	1.006	1.025	0.003
	Sex(Male)	0.710	0.524	0.962	0.027
	Region(Northern)	5.991	1.814	19.788	<0.001

^**a**^ estimated using Likelihood Ratio Tests

The model predicted that MDA participation in the previous year increased significantly with age (*χ*2 = 8.753, df = 1, p = 0.003). Males were significantly less likely to have taken MDA the previous year than females (*χ*2 = 6.25, df = 1, p = 0.027). Furthermore, those in the Northern Region were significantly more likely to have taken MDA the previous year than participants from the Western Region (*χ*2 = 15.407, df = 1, p = <0.001) with Region the strongest predictor of MDA participation included in the final model (OR = 5.991). There was no significant difference in MDA participation the previous year between those that slept under an LLIN the previous night and those that did not (*χ*2 = 21.815, df = 1, p = 0.072).

### Expected mf prevalence reduction in Agyan

**Fig 4:**
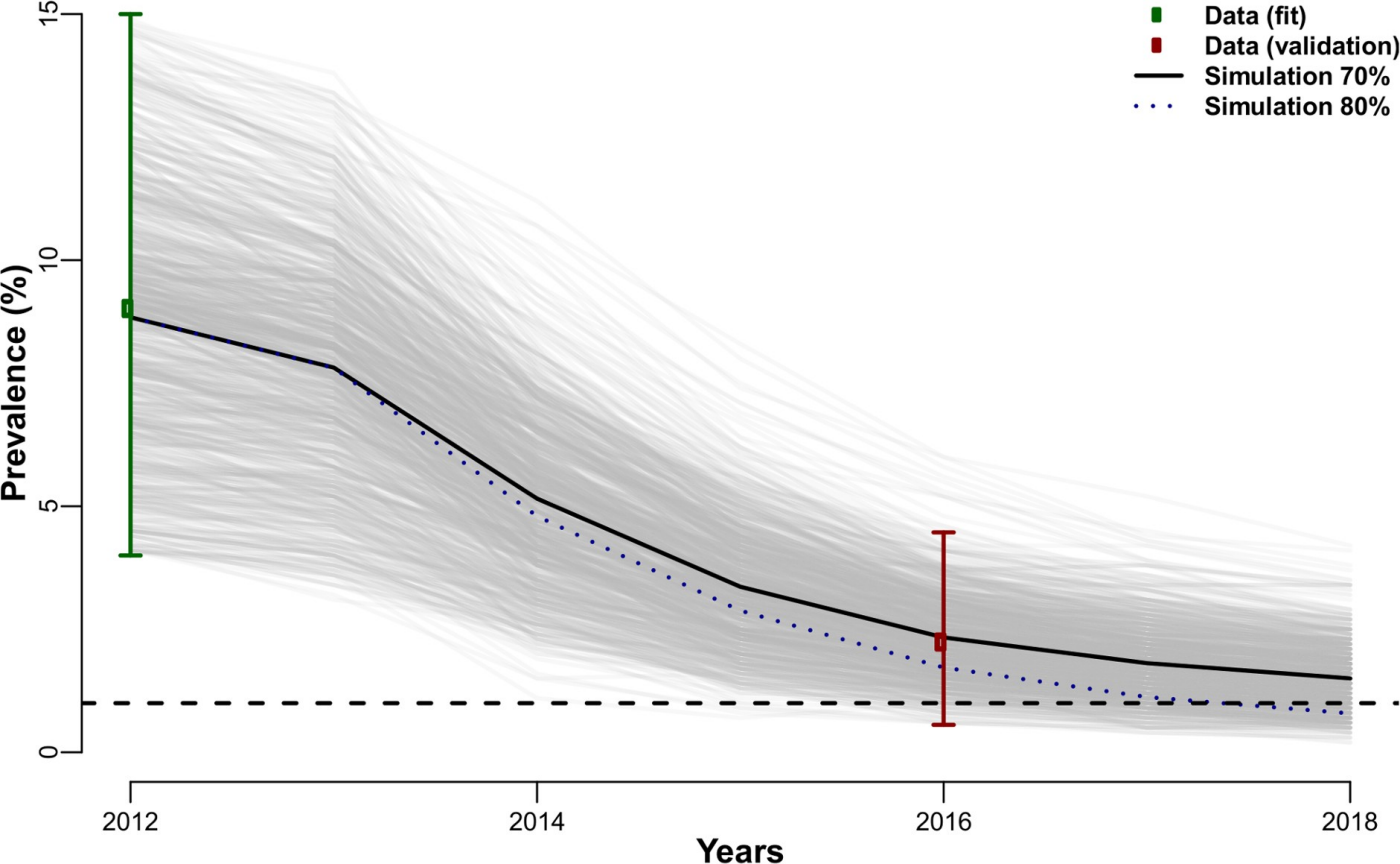
Observed and expected microfilaria prevalence reduction in Agyan province. Green and red circles represent the 2012 and 2016 prevalence estimates respectively, with their bootstrap confidence interval (green and red lines). The 2012 data were used to fit the model (only simulations in that prevalence range are used), while the 2016 data were used to validate the model (i.e. verify that most simulations fall within the red confidence interval). The grey lines are individual stochastic simulations (500 in total) with a 70% MDA coverage, median value shown as the black.

The observed and expected reduction in the mf prevalence across two time points (2012 to 2016) for which data were available are shown in Fig 4 under two scenarios with varying MDA coverage (80% and 70%). The simulations with TRANSFIL returned 5% of the stochastic simulations reaching the 1% elimination threshold in 2016 with 70% MDA coverage, this percentage of simulations increases to 22% if MDA coverage of 80% is assumed. Self-reported MDA coverage fell within this range although it is difficult to determine true coverage with a large proportion of participants unable to answer the question due to a lack of knowledge about the MDA program. The simulations show that an increase in coverage to 80% would have been required to reach the threshold by 2018.

The correlations in adherence between two consecutive rounds was estimated for a range of MDA coverage (20–90%). The maximum likelihood estimate for the adherence parameter *ρ* ranged 0.5–0.75 for the range of reported coverage (S1 Supporting Information).

## Discussion

Our results confirm the focal and rural nature of LF occurrence, with considerable variation in both antigen and microfilaria prevalence between communities and districts. As shown by previous surveys in Ghana [7,8], in the Northern Region a high and comparable prevalence of LF was observed in nearby rural villages in the savannah (Dugli and Sekyerekura, Nasoyiri and Seyiri) whereas no antigen positive individuals were detected in Jidanzana. In the surveyed communities in the Western Region, variation in prevalence was less evident but previous pre-MDA studies conducted in Ghana show the prevalence of LF varies greatly even at a small scale [26,27]. The prevalence of LF across Sub-Saharan Africa is highly variable [28], and the local environmental conditions influencing the occurrence and density of competent vectors play a major role in regional variation [29]. Furthermore, mf prevalence can fluctuate, and decreases have been observed also in the absence of MDA or other interventions [30].

Comparing our findings to the available historical data, it is clear that the mf prevalence has been declining over the years, possibly due to MDA. However, in most of the communities the prevalence was still above the target of 1% showing that further MDA rounds are still needed. The pre-control mf prevalence affects the duration of the intervention with a higher mf prevalence requiring more time to reach the elimination threshold [31]. The pre-MDA prevalence of mf in one of our studied communities, Agyan, in 1995 was 25.4% with a mean density of around 1,000 mf/ml of blood [26]. In the Nzema East district, in which Agyan is included, MDA started in 2004 [32] with 12 rounds of MDA completed in the area. Our 2016 data showed a prevalence of 2.0% and a mean mf density of 263 larvae/ml of blood. The results suggest that a starting high burden of infection will require a higher number of years of MDA to break transmission. As recently shown in India, pockets of LF transmission in areas previously classified as non-endemic can be easily overlooked without reliable mapping and enhanced surveillance [33]. It is possible that similar pre-control high burdens of infection were present in the other communities too, explaining the observed persistence of mf above the elimination threshold. This is also supported by our finding of a relatively high prevalence of filarial antigen in the adult population in the majority of our communities. Although in our surveys only the adult worm antigen was tested, a more sensitive and specific serological marker, such as antibodies produced against the infective L3 larvae-specific antigen Wb123 [34] would be needed to assess ongoing transmission. It is important to clearly distinguish between “hotspots for prevalence” (communities with a high infection burden pre-MDA) [11] and “hotspots for persistence” (communities with persistent infection due to factors other than the starting infection prevalence) to evaluate whether additional rounds of MDA are needed or more thorough investigations on why the program is failing.

In our study, antigen positivity was associated with increasing age and with being a male, yet was negatively associated with taking part in MDA the previous year. Males have been shown to have a significantly higher antigen and mf prevalence than females in Ghana [7,26,27], Cameroon [35], Sri Lanka [36] and Brazil [37], and the LF infection risk was also reported to be higher in males in a study from Congo [38]. In a case-control study conducted in Haiti comparing communities with persistent LF with LF-free communities after 6 years of MDA, systematic non-adherence with MDA emerged as the significant risk factor for LF persistence [39]. Not sleeping under a bed net is also a well-known risk factor for LF [37,40], and implementation of bednets can effectively reduce the number of LF infections in both humans and mosquitoes over time [19]. While our study identified no significant association between sleeping under a bed net the previous night and antigen positivity, the antigen is a marker of prior infection thus will not be immediately affected by MDA or bednet use. Other studies observed a lower but not significantly different antigen prevalence in people taking the MDA drugs and using bed nets compared to non-users [36], or the antigen prevalence was not different between groups of those adherent and non-adherent to MDA [41]. In a study from Congo, amongst males, those involved in hunting or fishing at night had a higher risk of being positive for LF antigen [38]. Interestingly, in the same study the use of bednets was protective for LF only for females. Our findings may confirm an increased risk of infection and LF persistence in males due to a lower MDA participation and exposure to outdoor biting during the night while fishing or performing other activities, particularly on the Western Region coastal villages, or when indoors, due to a lower or different use of bednets compared to females. In programmatic terms, these findings stress the importance of ensuring a better reach of the male population during MDA and LLIN distribution to ensure effective and sustained elimination. We recommend that the timing of the distribution activities should ensure the presence of all community members, and more gender-tailored sensitization activities on MDA and LLINs could be planned.

A significant effect of region was observed in relation to MDA participation and bednet ownership, with the Western Region communities showing lower rates of MDA participation and net ownership. Despite the reported LLIN ownership of >80%, the use of bednets in the surveyed communities was very low. We found an association between taking MDA and using a net. Furthermore, the major reason reported by the study participants for not participating in MDA or not owning a net was absence during the distribution. Overall, our findings indicate that a large portion of the community members are not reached by MDA and bednet distribution systematically, and this may disproportionately affect males. Similarly, our analyses suggest that the correlation in adherence is high, which would indicate that multiple rounds are almost always treating the same individuals. It is important to note however that our data have a very high recall bias with a disproportionate number of individuals recalling either 5 or 10 rounds (S1 Supporting Information).

Many logistical issues may interfere with adequate reach of all community members. These most commonly include difficulties in getting physical access to communities, particularly in rural settings, or inability of reaching community members within the allocated time [18]. In the Nzema East district in the Western Region, due to the major economic activities of mining, farming and fishing, most households cannot be reached during the day. Access during early morning or evening may not give enough time to reach all households, and distribution at night is not feasible in many communities which are not connected to the national electricity grid [18]. Reliable data on MDA program implementation are essential to assess the state of the program and its pitfalls. However, a recent study in Ghana showed that over 60% of the data regarding LF MDA, including demographics of the population treated and therapeutic and geographical coverage, are inaccurate [17]. In our study there was exceptionally high coverage reported by the program in the study villages, however we observed that nearly 30% of participants did not know about the program or had never taken drugs. There is a need to better understand the regional and district variations in logistics and data reporting which can affect the MDA effectiveness.

In addition to improvements in data collection, further qualitative research is needed. In particular, a combination of interviews and focus group discussions with both community members, health workers and drug distributors will help in identifying the factors within the national LF program, the community drug distributor networks, and at the household level that have hindered access or acceptance of the LF elimination program. For example, the training and motivation of community drug distributors to engage with community members and individual perceptions around the balance between the benefits and risk of adverse effects of treatment should be explored [16]. A qualitative approach would allow us to better understand the timing and seasonality of community activities throughout the year, which is pivotal to the planning of effective MDA and LLIN distributions. An equitable LF program will ensure that the identified solutions take into account how gender, age, religion and socioeconomic status intersect in refining their approach [42].

Great benefits for the elimination program would also come from a better understanding of gender-specific behaviours and attitudes towards complementary interventions such as LLINs, with attention to the potential geographic variability. In parallel with MDA, high LLIN coverage coupled with other vector control interventions in an integrated vector management approach, would play an important role in sustaining elimination and avoiding resurgence in the eventuality of the re-introduction of the parasite through human migration from other endemic areas [15]. However, as also confirmed by our findings, it is pivotal to support increased bednet usage through better communication during net distribution [43]. Furthermore, outdoor biting by anopheline vectors, for which LLINs offer no protection, must not be discounted and there is a need for more detailed entomological studies addressing local vector and human behaviours. Our preliminary data from the Western Region coastal villages suggest *A*. *melas* to be the most abundant vector. Previous studies conducted in this part of Ghana showed that this species frequently bites outdoors, and many people in these settings sleep outdoors [44]. If further MDA and bednet distributions are planned in this region, efforts should be made to reach high-risk groups and other systematically non-adherent members of the community.

Given the availability of historical data for the community of Agyan, we used the TRANSFIL model to verify whether the mf prevalence observed in this study could be predicted given the values of MDA coverage collected in the survey and whether variation in coverage could help explain the persistence of mf above the 1% threshold to scale down MDA. Our results showed that the currently observed prevalence in Agyan is within the range we would expect based on the 2012 prevalence, and an increase in MDA coverage is necessary to accelerate the decline. There are limitations to such analysis, the starting mf prevalence used in the model was not the true pre-MDA value since in 2012 the MDA was already in its 8^th^ year, and the values for MDA coverage are fixed so variation over time (as an effect of the MDA scaling up) is not accounted for. Despite these limitations, our results show that the TRANSFIL model can be used to simulate mf reductions and program achievements in varying conditions of intervention coverage using data collected over time in the field. Our results suggest that improvements to the MDA program should focus on ensuring adequate coverage, particularly of the individuals that have been systematically missed during the distribution due to occupational commitments. Our study has highlighted regional, gender and age-based differences in intervention access and adherence due to availability at the time of the distribution. These findings have led to a commitment within the National LF elimination program to apply the Quality Improvement (QI) research strategy to work with frontline health workers and develop a plan to address bottlenecks in coverage. This includes a pre- and post-intervention assessment of coverage to evaluate improvements and revise the timing, frequency and the community engagement activities in hotspot districts. Timelines for the distribution should weigh socioeconomic constraints and caring responsibilities of community members with donor requirements and budgetary demands.

Our findings suggest that the current persistence of microfilaria in communities still under MDA is likely to be due to the pre-MDA high burden of infection and systematic non-adherence despite adequate MDA coverage. Although progress towards the 1% mf prevalence threshold has been slower than other regions, our modelling results show that the current prevalence is what we would expect based on specific community parameters. Model results also suggest that MDA coverage closer to 80% is needed to cross that threshold in the near term. Our study has highlighted where gaps in intervention adherence lie, and confirmed the individuals not accessing interventions are more likely to be infected. Reaching the community members which may still constitute reservoirs of infection is important to not only reach but also sustain LF elimination following TAS evaluation and the scale down of MDA. It is pivotal to address the issues limiting effective reach of all eligible community members during MDA and LLIN distribution, and to understand the factors underlying community-wide and individual MDA adherence, net use and exposure to LF vectors using an integrated quantitative and qualitative research approach. Addressing all these aspects will help Ghana reach the 2020 elimination goal, and will also be beneficial to all the countries striving to stop LF transmission and scale down MDA.

## Supporting information

S1 Checklist STROBE checklist. (DOCX)

S1 Supporting Information TRANSFIL model implementation methodology and outputs. (PDF)

S1 Table Filarial antigen prevalence by gender, age group and community. (DOCX)
