# Supplementary material for: Elimination within reach: A cross-sectional study highlighting the factors that contribute to persistent lymphatic filariasis in eight communities in rural Ghana
Source: PLoS Negl Trop Dis. 2019 Jan 4;13(1):e0006994. doi: 10.1371/journal.pntd.0006994 (PMC6342320; doi:10.1371/journal.pntd.0006994)
Supplement: S1 Supporting Information — (PDF) [file pntd.0006994.s002.pdf]

S2 Supporting information - TRANSFIL implementation methodology

June 7, 2018

We implemented the most up-to-date version of TRANSFIL, described in detail in [1]. Briefly, it is an stochastic individual based transmission model of Lymphatic filariasis in a population. The model reproduces the life cycle of filarial worm in the human host, as well as transmission between individuals through the vector (in this particular setting, *Anopheles* mosquitoes). The model has been calibrated in the past to real data [1, 2], the fixed parameters are not discussed further here. Implementation in the Agyan community is described below.

1 Baseline prevalence in 2012

A study was done in the Agyan community in 2012, where 9 individuals, out of 100 individuals tested, were positive for microfilarial worms (Ghana Health Service, unpublished). Assuming that this study is representative of the whole population, we used bootstrapping to estimate confidence intervals (CI) for the value of prevalence and obtained a CI range $4\%-15\%$, figure S2.1.

We initialized multiple runs, with a range of prevalence values in 2012 and selected 500 simulations that reproduced the distribution of prevalence in 2012 within the confidence interval obtained from the bootstrap. These 500 runs were used for the analysis in the forward simulations.

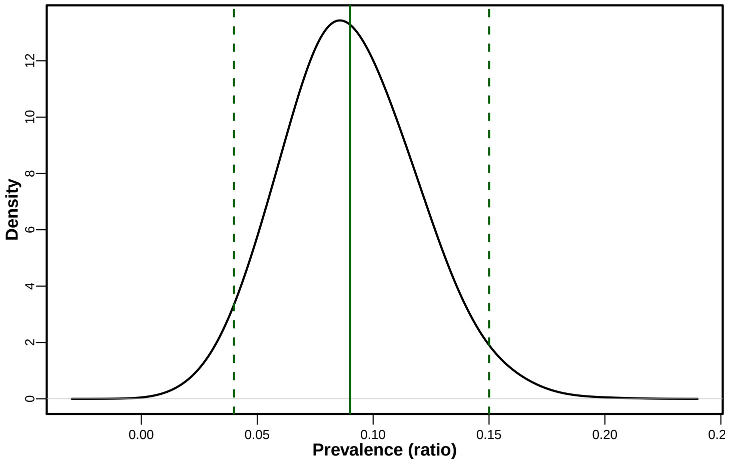

Figure S2.1: Density plot of the prevalence in 2012 estimated by bootstrapping, mean denoted with the solid green line; dashed green lines indicate the 95% quantiles. A gaussian kernel was used with a window size of 0.01.

2 Forward simulations

To parametrize the forward simulations, we need information on the coverage and adherence of the MDA rounds. We refer here to coverage as the proportion of the population treated and adherence as the correlation for individuals being treated two consecutive rounds [3].

We considered a range of annual MDA coverage (from 20% to 90%), for a total of 36 different values considered. We modelled individuals' adherence after multiple rounds of treatment based on the paper by Griffin *et al.* [4], alternative approaches to model adherence have been reviewed recently by Dyson *et al.* [3], with the approach by Griffin the one currently implemented in TRANSFIL [1]. In this approach, a parameter ρ is used to model the probability of an individual making the same decision than in the previous round of treatment. We selected the ρ values for our simulations by using maximum likelihood to minimize the difference in the distribution of the number of rounds attended after ten treatments compared to our data [ref here]. We estimated the ρ value for a range of coverage (from 20% to 90% - total of 36 different coverage values), figure S2.2. It is important to note, that although we are fitting by maximum likelihood, the fit is not perfect, due to the large amount of recall bias, with high peaks in the number of

individuals reporting 5 and 10 treatments (26.1% and 38.7% respectively).

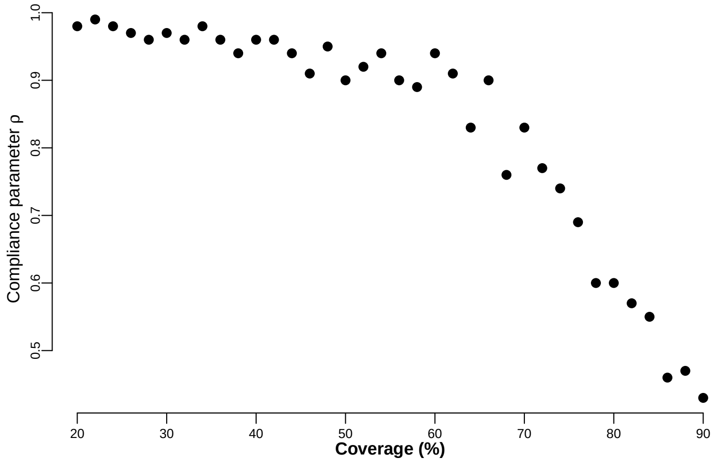

Figure S2.2: Maximum likelihood estimate of the adherence parameter ρ, for a range of MDA coverage.

3 Prevalence in 2016

We estimated the prevalence in 2016 with a survey where we found four positives out of 179 individuals tested (2.2% prevalence). Similarly to the prevalence in 2012, we can calculate the confidence interval through bootstrapping (CI range 0.5%−4.4%), figure S2.3.

We then calculated the coverage that would better fit the prevalence that we estimated from the survey. This was done by minimizing the squared error in the estimation of prevalence in 2016, between the 500 simulations and the value obtained from the survey (2.2%), for each value of coverage considered (range from 20% to 90%), figure S2.4. This assumes coverage does not change over time. The smallest squared error is achieved with a coverage of 76%, which suggest this was the most likely coverage. It is important to note however that the squared errors in the range between 70% (recently estimated coverage by Ghana Health Service) and 80% (coverage reported by the Ministry of Health) are very similar.

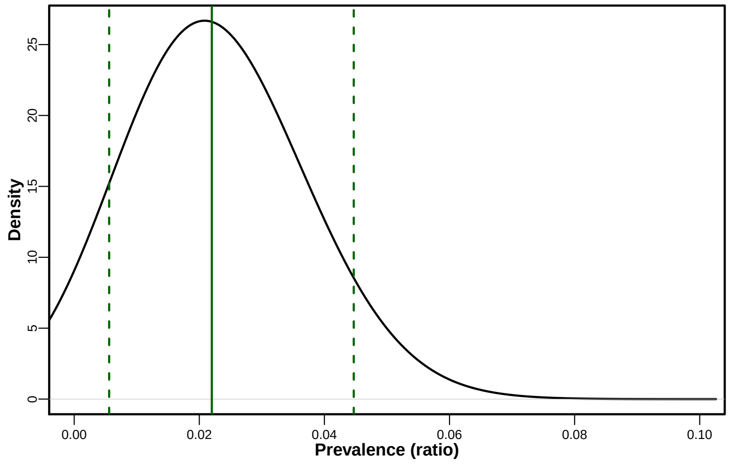

Figure S2.3: Density plot of the prevalence in 2016 estimated by bootstrapping, mean denoted with the solid green line; dashed green lines indicate the 95% quantiles. A gaussian kernel was used with a window size of 0.01.

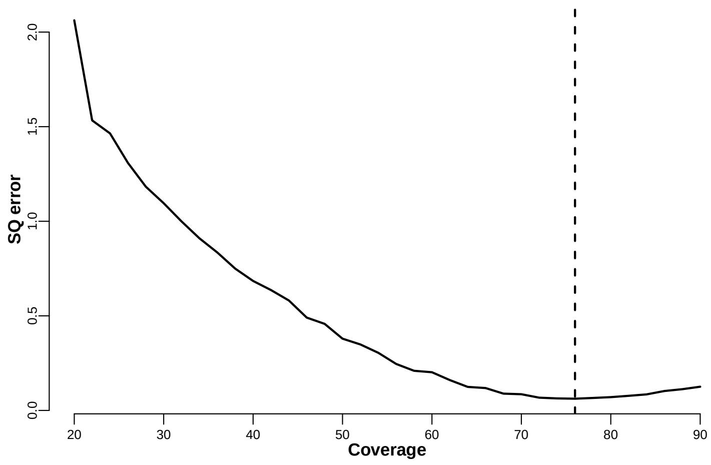

Figure S2.4: Least squares error in the estimation of prevalence in 2016 for different values of coverage. Minimum error is achieved with a coverage of 76%.

References

[1] M. Irvine, L. Reimer, S. Njenga, S. Gunawardena, L. Kelly-Hope, M. Bockarie, and T. Hollingsworth, "Modelling strategies to break transmission of lymphatic filariasis - aggregation, adherence and vector competence greatly alter elimination," *Parasites & Vectors*, vol. 8, no. 1, p. 547, 2015.

[2] M. E. Smith, B. K. Singh, M. A. Irvine, W. A. Stolk, S. Subramanian, T. D. Hollingsworth, and E. Michael, "Predicting lymphatic filariasis transmission and elimination dynamics using a multi-model ensemble framework," *Epidemics*, vol. 18, pp. 16–28, 2017.

[3] L. Dyson, W. Stolk, S. Farrell, and T. Hollingsworth, "Measuring and modelling the effects of systematic non-adherence to mass drug administration," *Epidemics*, vol. 18, pp. 56–66, 2017.

[4] J. T. Griffin, T. D. Hollingsworth, L. C. Okell, T. S. Churcher, M. White, W. Hinsley, T. Bousema, C. J. Drakeley, N. M. Ferguson, M.-G. Basez, and A. C. Ghani, "Reducing Plasmodium falciparum malaria transmission in Africa: A model-based evaluation of intervention strategies," *PLOS Medicine*, vol. 7, no. 8, pp. 1–17, 2010.
