# Supplementary material for: Elimination within reach: A cross-sectional study highlighting the factors that contribute to persistent lymphatic filariasis in eight communities in rural Ghana
Source: PLoS Negl Trop Dis. 2019 Jan 4;13(1):e0006994. doi: 10.1371/journal.pntd.0006994 (PMC6342320; doi:10.1371/journal.pntd.0006994)
Supplement: S1 Table — (DOCX) [file pntd.0006994.s003.docx]

|  | | **% FTS + (no. +/no. tested)** | | | | | | |
| --- | --- | --- | --- | --- | --- | --- | --- | --- |
| **Community** | **Sex** | **18-20y** | **21-30y** | **31-40y** | **41-50y** | **51-60y** | **60+y** | **Overall** |
| Agyan | Male | 18.2 (2/11) | 20.7 (6/29) | 56.3 (9/16) | 46.7 (7/15) | 0 (0/6) | 60 (3/5) | 32.9 (27/82) |
|  | Female | 9.1 (1/11) | 16.1 (5/31) | 18.8 (3/16) | 16.7 (1/6) | 21.4 (3/14) | 18.2 (2/11) | 16.6 (15/89) |
| Ampain | Male | 0 (0/11) | 12.5 (3/24) | 27.8 (5/18) | 12.5 (1/8) | 25 (1/4) | 0 (0/10) | 13.3 (10/75) |
|  | Female | 10 (1/10) | 0 (0/9) | 0 (0/21) | 0 (0/14) | 11.1 (1/9) | 9.1 (2/22) | 4.7 (4/85) |
| Sanwoma | Male | 14.3 (1/7) | 12.5 (2/16) | 25 (3/12) | 9.1 (1/11) | 33.3 (1/3) | 0 (0/4) | 15.1 (8/53) |
|  | Female | 6.7 (1/15) | 7.1 (3/42) | 6.3 (2/32) | 21.4 (3/14) | 0 (0/9) | 9.1 (1/11) | 8.1 (10/123) |
| Dugli | Male | 16.7 (1/6) | 25.9 (7/27) | 6.7 (1/15) | 33.3 (2/6) | 75 (3/4) | 30 (3/10) | 25 (17/68) |
|  | Female | 0 (0/10) | 28.6 (6/21) | 9.1 (1/11) | 13.3 (2/15) | 10 (1/10) | 20 (1/5) | 15.3 (11/72) |
| Sekyerekura | Male | 37.5 (3/8) | 27.3 (3/11) | 22.2 (2/9) | 0 (0/6) | n/a | 0 (0/1) | 22.9 (8/35) |
|  | Female | 0 (0/2) | 12.5 (2/16) | 13.3 (2/15) | 66.7 (2/3) | 0 (0/2) | n/a | 15.8 (6/38) |
| Nasoyiri | Male | 0 (0/9) | 16.7 (2/12) | 37.5 (3/8) | 33.3 (1/3) | 0 (0/6) | 0 (0/4) | 14.3 (6/42) |
|  | Female | 11.1 (1/9) | 0 (0/12) | 9.1 (1/11) | 25 (4/16) | 35.7 (5/14) | 0 (0/6) | 16.2 (11/68) |
| Seyiri | Male | 12.5 (1/8) | 20 (3/15) | 14.3 (1/7) | 66.7 (2/3) | 100 (3/3) | 50 (2/4) | 30 (12/40) |
|  | Female | 14.3 (1/7) | 40 (4/10) | 36.4 (4/11) | 37.5 (3/8) | 0 (0/4) | 0 (0/2) | 28.6 (12/42) |
